# Non-structural carbohydrate concentrations in tree organs vary across biomes and leaf habits, but are independent of the fast-slow plant economic spectrum

**DOI:** 10.3389/fpls.2024.1375958

**Published:** 2024-05-03

**Authors:** Jorge Andres Ramirez, Dylan Craven, David Herrera, Juan Manuel Posada, Bjorn Reu, Carlos A. Sierra, Guenter Hoch, Ira Tanya Handa, Christian Messier

**Affiliations:** ^1^Facultad de Ciencias Agrarias, Universidad del Cauca, Popayán, Colombia; ^2^Centre d’Étude de la Forêt (CEF), Université du Québec à Montréal, Montréal, QC, Canada; ^3^GEMA Center for Genomics, Ecology and Environment, Universidad Mayor, Huechuraba, Santiago, Chile; ^4^Data Observatory Foundation, and Technology Center, Santiago, Chile; ^5^Department of Biogeochemical Processes, Max Planck Institute for Biogeochemistry, Jena, Germany; ^6^Department of Biology, Faculty of Natural Sciences, Universidad del Rosario, Bogotá, Colombia; ^7^School of Biology, Universidad Industrial de Santander, Bucaramanga, Colombia; ^8^Max Planck Institute for Biogeochemistry, Jena, Germany; ^9^Department of Environmental Sciences – Botany, University of Basel, Basel, Switzerland; ^10^Institut des Sciences de la Foret Tempérée, Université du Québec en Outaouais, Ripon, QC, Canada

**Keywords:** carbohydrate reserves, trait syndromes, leaf habit, carbon investment strategies, tropical trees, temperate trees

## Abstract

Carbohydrate reserves play a vital role in plant survival during periods of negative carbon balance. Under a carbon-limited scenario, we expect a trade-offs between carbon allocation to growth, reserves, and defense. A resulting hypothesis is that carbon allocation to reserves exhibits a coordinated variation with functional traits associated with the ‘fast-slow’ plant economics spectrum. We tested the relationship between non-structural carbohydrates (NSC) of tree organs and functional traits using 61 angiosperm tree species from temperate and tropical forests with phylogenetic hierarchical Bayesian models. Our results provide evidence that NSC concentrations in stems and branches are decoupled from plant functional traits. while those in roots are weakly coupled with plant functional traits. In contrast, we found that variation between NSC concentrations in leaves and the fast-slow trait spectrum was coordinated, as species with higher leaf NSC had trait values associated with resource conservative species, such as lower SLA, leaf N, and leaf P. We also detected a small effect of leaf habit on the variation of NSC concentrations in branches and roots. Efforts to predict the response of ecosystems to global change will need to integrate a suite of plant traits, such as NSC concentrations in woody organs, that are independent of the ‘fast-slow’ plant economics spectrum and that capture how species respond to a broad range of global change drivers.

## 1 Introduction

Carbon allocation to growth is a fundamental process that underpins global variation in plant functional traits, which describe a gradient from resource acquisitive to resource conservative species (Grime et al., 1997; Diaz et al., 2004; Wright et al., 2004; Chave et al., 2009; Reich, 2014; Diaz et al., 2016). These trade-offs reflect variation among plant traits for species that differ in growth form, size, and evolutionary history (Reich et al., 1997, 1999; Wright et al., 2004; Donovan et al., 2011; Reich, 2014; Diaz et al., 2016). For example, fast-growing, resource-acquisitive species, typically have high specific leaf area (SLA), high leaf nutrient concentrations, and low wood density (hereafter ‘fast’ species). In contrast, slow-growing, resource-conservative species, are characterized by low SLA, low leaf-nutrient concentrations, and high wood density (hereafter ‘slow’ species). While ‘slow’ trait values imply high construction costs, they may also allow trees to enhance resilience to different biotic or abiotic stress factors (Coley et al., 1985; Poorter and Kitajima, 2007).

Trees may exhibit different trait values as an adaptation to environmental conditions that limit growth (e.g., water availability and average growth temperature) along environmental gradients (Swenson and Enquist, 2007; Cornwell and Ackerly, 2009; Read et al., 2014; Wieczynski et al., 2019) and, thus, exhibit different non-structural carbohydrates (NSC) concentrations. There is growing evidence that trees increase the storage of NSC and N in winter, and increase SLA and photosynthetic rates during the growing season as an acclimation strategy for maintaining metabolic activity in colder environments (Tjoelker et al., 1999; Campbell et al., 2007; Xiang et al., 2013). In addition, temperate species with high SLA and high photosynthetic rates likely contain tightly packed palisade parenchyma cells in which NSC are stored (Poorter et al., 2010) and, thus, may accumulate reserves rapidly in woody tissues for use during the dormant season and for bud break the following growing season (Kramer and Kozlowski, 1979). Another factor that may influence the relationship between traits and leaf NSC concentrations in leaves is that, independent of whether a species is ‘slow’ or ‘fast’ in terms of carbon use, leaf damage by herbivory and pathogens may be higher in warmer and more humid habitats where leaf dry matter content and leaf size tend to be higher than in deciduous temperate forests (Niinemets et al., 2007; Zhang et al., 2017). Thus, tropical species may invest preferentially more carbon in structural leaf defenses than in carbohydrate reserves because carbon allocation to both may not be possible under conditions of limiting resources.

The storage and mobilization of NSC may increase tree survival and recovery during periods of negative carbon balance (i.e. drought, herbivory, etc.) (Kobe, 1997; Canham et al., 1999; Myers and Kitajima, 2007; Poorter and Kitajima, 2007; Atkinson et al., 2014; O’Brien et al., 2014). Thus, a significant fraction of the carbon captured by photosynthesis is allocated to carbohydrate reserves in the form of NSC (Hoch and Körner, 2003; Hoch et al., 2003; Landhäusser and Lieffers, 2003; Würth et al., 2005; Piper et al., 2009; Martínez-Vilalta et al., 2016). In general, NSC are comprised of low weight sugars and starch (Hoch et al., 2003). Sugars are mobilized easily and used for short-term storage (i.e. within a growing season), while starch is stored in a more recalcitrant form for long-term use (Chapin et al., 1990; Dietze et al., 2014; Hartmann and Trumbore, 2016; Martínez-Vilalta et al., 2016).

For most deciduous species, NSC support physiological activity during dormant periods and the flushing of new leaves (Newell et al., 2002; Gaucher et al., 2005; Würth et al., 2005; Gough et al., 2009; Messier et al., 2009; Fajardo et al., 2013; Klein et al., 2016). In contrast, most evergreen species accumulate NSC in their tissues throughout the year (Hoch et al., 2003). Additionally, NSC may increase resilience to natural or anthropogenic disturbances, providing the energy and substrates for the vital functions of plants (i.e. growth, defense, reproduction, resprouting, and survival) (Chapin et al., 1990; Kozlowski, 1992; Dietze et al., 2014; O’Brien et al., 2020). Availability of NSC may drastically reduce the risk of mortality by supplying carbon to metabolism during droughts (O’Brien et al., 2014; Doughty et al., 2015; Rowland et al., 2015; O’Brien et al., 2020; Piper and Paula, 2020), which are becoming increasingly common across biomes. Additionally, other common disturbances in forest ecosystems, such as fires, windstorms, ice storms, and insect outbreaks, may favor species that maintain high concentrations of NSC (Dietze et al., 2014; Hartmann and Trumbore, 2016; Martínez-Vilalta et al., 2016). Consequently, we expect that evergreen and deciduous species will exhibit contrasting patterns of NSC accumulation across forest biomes.

NSC concentrations increase via accumulation of reserves (Chapin et al., 1990). NSC reserve formation is a ‘passive’ process driven by the imbalance between photosynthesis supply and the demand of carbon for growth and respiration (Chapin et al., 1990; Sala et al., 2012; Dietze et al., 2014). NSC reserve formation can also be an active process when carbon supply is limited (i.e. stomatal closure). Thus, NSC reserve formation may be both an active and a passive process, but is context-dependent (Litton et al., 2007; Dietze et al., 2014; Weber et al., 2018). It remains uncertain whether the ‘fast-slow’ plant economics spectrum (Reich, 2014), which captures variation in life-history strategies, varies in coordination with NSC concentrations in leaves and woody organs. Under a carbon-limited scenario, a trade-off between carbon allocation to growth and to reserves and defense is suggested (Kitajima, 1994; Kobe, 1997; Myers and Kitajima, 2007). Thus, tough leaves and dense woody organs suggest greater carbon investment in defense traits to resist and to recover from biotic and abiotic stress (Poorter and Kitajima, 2007; Poorter et al., 2010), which co-vary with carbon allocation to reserves, especially in roots (Kitajima, 1994; Myers and Kitajima, 2007). Also, since a higher SLA indicates a higher light capture potential, a higher net photosynthetic rate, and higher concentrations of foliar nutrients such as N (Wright et al., 2002, 2004), an increase in SLA may lead to an increase in the proportion of metabolically active carbon allocated to growth of woody organs (Shipley et al., 2006; Li et al., 2016), which may lead to less storage in fast growing species. However, high levels of NSC concentrations have been also associated with resource-acquisitive species, as high levels of remobilized resources allow for high growth rates (Uscola et al., 2015). However, life history traits such as leaf habit (evergreen or deciduous), which evolved as an adaptation to freezing temperatures (Zanne et al., 2014), could mediate the relationship between the fast-slow plant economics spectrum and NSC concentrations. For example, because of their slower, yet less variable photosynthetic rates, evergreen tree species may exhibit weaker or contrasting trait-NSC relationships across organs than deciduous tree species.

An alternative hypothesis is that NSC concentrations are decoupled from, or are orthogonal to, the ‘fast-slow’ plant economics spectrum. This pattern suggests that variation in NSC concentrations are uncorrelated with effect traits, which are associated with species’ effects on ecosystem functioning. It may form part of an independent axis of ecological variation including a broader set of response traits whose diversity may play a role in determining the resilience of ecosystems to global change (Suding et al., 2008; Mori et al., 2013). We would expect, therefore, that NSC concentrations to be more strongly correlated with traits relating to growth rate, survival, or reproduction, such as adult plant size, wood density, drought, shade, and waterlogging tolerance, seed mass, mode of reproduction (vegetative or sexual), and seed dispersal vector, than traits relating to ecosystem processes, such as N cycling or C storage (Violle et al., 2007; Suding et al., 2008; Brousseau et al., 2018). The extent to which a trait-based spectrum of resilience is generalizable is of basic and applied importance as it will contribute towards improving predictions of how ecosystem functioning responds to global change.

We therefore examine how the relative contributions of biome, leaf habit, and species to the variation in NSC concentrations for each tree organ vary with key functional traits in angiosperm tree species, a central issue for predicting the role of NSC in the resilience of trees that differ in life strategies. Here, we test the hypothesis that, once accounting for phylogenetic relationships among species (Freckleton et al., 2002), NSC concentrations in woody organs and leaves will be coordinated with plant functional traits that underpin the ‘fast-slow’ plant economics spectrum across biomes. Further, we anticipate that species with ‘slow’ traits associated with greater carbon investment in defense and conservative ecological strategies, such as a low SLA, high tissue density, and low concentrations of leaf nutrients will accumulate more NSC in woody organs (stem, branch, and root) than species associated with acquisitive or ‘fast’ ecological strategies. Because of differences in leaf phenology, evergreen tree species may exhibit contrasting relationships between the ‘fast-slow’ plant economics spectrum and NSC concentrations across tree organs.

## 2 Materials and methods

### 2.1 Research sites

We performed this study in a deciduous temperate forest (DTF; Mont St-Hilaire, Quebec, Canada) and in an upper montane tropical forest (UMF) and a lowland tropical forest (LTF) in Colombia (**Supplementary information**, **Supplementary Table 1**). These three sites were selected for their contrasts in latitude, seasonality (temperate versus tropical), and elevation (lowland and upper montane forests (Colombia) (**Supplementary Figure 1**). Each study site within each biome were protected and did not experienced recent anthropogenic disturbances (at least during the last 20 years). In the LTF, climate does not exhibit marked seasonality in terms of temperature and precipitation. The climate in the UMF exhibits a bimodal variation of precipitation between the rainy and dry seasons; the first dry period lasts from November to March, while the second one from June to August. In contrast, the climate in the DTF is characterized by strong intra-annual variation in temperature, with average sub-zero temperatures from November to March, mild and wet summers (June-September) and a growing season from May to October (**Supplementary Figure 1**).

### 2.2 Field sampling

We sampled a total of 61 native tree species (see species list and leaf habit in **Supplementary Table 2**) across the three sites in 2012. In both biomes, we sampled when we expected NSC concentrations to be highest, as the objective of our study is to test trait-NSC relationships across biomes and species (Herrera-Ramírez et al., 2021). In the temperate forest site we sampled towards the end of the growing season (October) and in the tropical forest sites (LTF and UMF) during the rainy season (January to April). At each site, we selected abundant tree species for sampling. In Colombia, tree species were selected by consulting with researchers familiar with local ecosystems to have a representative sample of the plant communities since no biomass or abundance data were available. In Quebec, species were selected based on abundance data of Mont St. Hilaire (Maycock, 1961; Arii et al., 2005), which is why we did not sample evergreen tree species in the DTF. At the tropical forest sites, we sampled evergreen and deciduous species. Botanical samples of all tropical species were verified and deposited at the Medellín Botanical Garden Herbarium Joaquin Antonio Uribe (JAUM).

Tree diameter at breast height and height were measured for all sampled individuals. Leaves and woody organs (branches, stems, and roots) were sampled from 3-5 individuals for each species. Current year leaves from adult plants without visible symptoms of pathogen or herbivore attack were sampled. To avoid possible effects of diurnal variation in NSC, leaf samples were collected in the early morning (Upmeyer and Koller, 1973; Pérez-Harguindeguy et al., 2013). Leaf samples were taken from one sun-lit branch at the top of the canopy with a tree trimmer or by climbing the trees, and then divided in two groups. One group was placed in paper bags for NSC measurements, while the second group was placed in plastic bags with damp tissue for measurement of leaf traits (see below). Stem samples were taken with a 4.3 mm diameter increment borer. Stem cores were taken perpendicular to the slope to reduce variability in wood density due to compression or tension. Samples of sun-lit branches 2-3 cm in diameter were obtained by cutting them with a tree trimmer. Root samples were taken with an increment borer from large surface roots ca. 50 cm away from the base of the stem. All samples for NSC analysis were put in paper bags and then in a cooler. In total, we collected and analyzed samples from 326 trees.

### 2.3 Non-structural carbohydrates (sugar, starch, and NSC, % of dry matter)

All NSC samples were microwaved within 8 h after sampling to stop enzymatic activity (Popp et al., 1996; Landhäusser et al., 2018). Leaf samples were ground using a ball mill and wood samples using a coffee grinder with a mesh sieve. Due to the large number of samples available, we selected 180 sub-samples (of a total of 1,271 samples) using the Kennard**–**Stone algorithm (Kennard and Stone, 1969) for NSC analysis following Hoch et al. (2002) based on variation in near-infrared spectra. Ground plant material was dissolved for 30 min in distilled water. Starch and sucrose were disaggregated in glucose and in glucose and fructose, respectively, with Clarase (*Aspergillus oryzae*, Enzyme Solutions Pty Ltd, Crydon South, Victoria, Australia) by incubation at 40˚C for 15 h. Phosphoglucose-isomerase was added to the solution and then the total amount of glucose (corresponding to total NSC) was quantified photo-metrically in a microplate photometer at 340 nm (Thermo Fisher Scientific, Waltham, USA) after conversion of glucose to gluconate-6-phosphate (hexokinase; Sigma-Aldrich, St. Louis, MO, USA). An aliquot of the original extract was treated with invertase and phosphoglucose-isomerase (both Sigma-Aldrich) to breakdown all soluble sugars into glucose. We followed the approach of Hoch et al. (2003) for calculating starch content by subtracting sugar from NSC. We used pure starch and solutions of glucose, fructose, sucrose, and plant powder (orchard leaves; Leco, St. Joseph, MI, USA) as standards and to control reproducibility of the extraction.

Using the same dataset, we extrapolated NSC values from the 180 sub-samples to all samples using near-infrared reflectance spectra (**Supplementary Figure 2**; Ramirez et al., 2015). Reflectance spectra were measured using a FT-NIR spectrometer Analyzer (Bruker MPA Multi-Purpose FT-NIR Analyzer, Bruker Optik GmbH, Ettlingen, Germany) for all samples. The reflectance spectra were taken from 800 nm to 2780 nm with a mean spectral resolution of 1.7 nm on five scans per sample. The spectral data were recorded as absorbance (log (1/R), where R = reflectance). Then, we fitted regression models that predict NSC concentrations in different tree organs (leaves, branches, stems, and roots) from near-infrared reflectance spectra using partial least squares regression and competitive adaptive re-weighted sampling (Li et al., 2009). Across all tree organs, the model fit for NSC was *r*^2 = ^0.91 (Ramirez et al., 2015). The NSC concentrations are reported as the percentage of dry matter.

Because tree height may influence NSC allocation patterns (Sala and Hoch, 2009; Genet et al., 2010; Piper and Fajardo, 2011; Woodruff and Meinzer, 2011), we selected the tallest trees of each species (4 – 31 m). All sampled trees were at or close to their maximum height as they were sampled in mature forests. Tree height was measured with a TruPulse 360 laser with a resolution of 10 cm for linear lengths (Laser Technology, Inc., CO, USA).

### 2.4 Functional traits

We measured 11 effect traits that are associated with important ecological strategies for tree functioning, productivity, and survival (**Supplementary Table 3**) following standard protocols (Pérez-Harguindeguy et al., 2013).

#### 2.4.1 Leaf size (mm^2^), leaf thickness (mm), leaf dry matter content (mg g^-1^), and specific leaf area (mm^2^ mg^-1^)

Eight completely expanded leaves were randomly collected from all the leaves of a sampled branch for each individual tree. Leaves were placed in plastic bags in the field with damp paper to maintain humidity. After determining fresh leaf mass, we dried leaf samples in an oven at 60°C until samples reached constant weight. LS was measured using WinFolia (Regent Instruments, Toronto, Canada). LT was measured on fresh leaves as the mean of four measurements with a digital micrometer (Mitutoyo Instruments, Singapore). LDMC was calculated as leaf dry mass divided by its fresh saturated mass and SLA was calculated as the area of the fresh lamina surface divided by its dry mass.

#### 2.4.2 Photosynthetic capacity by mass (*A*_mass_, nmol CO_2_ g^-1^ s^-1^)

Photosynthetic capacity was measured on six leaves from two sun-lit branches in both tropical forest sites using a LI-6400 portable photosynthesis system (LI-COR, Lincoln, NE, USA). Branches for photosynthesis determination were placed in a bucket of water during the measurements to avoid disruption of water transport within the xylem (Verryckt et al., 2020). Leaves were light adapted to reduce the chances that leaves would close their stomata due to an abrupt increase in PPFD and then the photosynthetic capacity under saturating light (*A*_max_) was measured at 2000 µmol m^-2^ s^-1^. Measurements were carried out under ambient CO_2_ concentrations (390 ppm) as recommended by (Pérez-Harguindeguy et al., 2013), and leaf temperature (set at 20°C). Photosynthetic data for tree species in the DTF were obtained from (Marino et al., 2010), which were measured in a similar manner as described above.

#### 2.4.3 Leaf elements (leaf N (%) and leaf Ca, leaf Mg, and leaf P (mg kg^-1^)

About 20 g of leaf tissue per tree were dried and ground to a fine powder using a ball mill. Nitrogen concentrations were determined for all leaf samples with a CN elemental analyzer (Vario MAX, Elementar, Germany). Determination of Ca, Mg, and P was performed on 100 samples using the acid digest method (Allen et al., 1974), and these results were extrapolated to all leaf samples using FT-NIR reflectance spectroscopy as described for NSC (Ramirez et al., 2015). Model fit for leaf nutrients was (*r*^2^) 0.93, 0.76 and 0.78 for Ca, Mg, and P, respectively (**Supplementary Figure 2**).

#### 2.4.4 Wood density (stem density and branch density (BD), mg mm^-3^)

Samples of stems and branches were placed in plastic bags in the field with damp paper to maintain humidity, and then were soaked in water in the lab for 48 hours. Fresh wood volume was measured without bark by water displacement, and wood mass was determined after drying samples at 60°C, and then again at 100°C, to a constant weight (Williamson and Wiemann, 2010).

### 2.5 Statistical analysis

#### 2.5.1 Phylogeny

We used an updated version of the molecular phylogeny from Zanne et al. (2014) and Qian and Jin (2015) to build a phylogeny with the *congeneric.merge* function in the ‘*pez*’ R package (Pearse et al., 2015), conservatively binding species into the backbone using dating information from congeners in the tree.

#### 2.5.2 ‘Fast-slow’ spectrum

We performed a principal components analysis using the PCA function in the R package “*FactoMineR*” (Lê et al., 2008) to represent the *‘*fast-slow*’* spectrum of plant form and function (Diaz et al., 2016). Prior to analysis, leaf area, leaf thickness, SLA, and *A*_mass_ were natural log transformed to meet normality assumptions and all traits were standardized using a z-transformation. Because the first two axes of the PCA (PC1 and PC2) explain a considerable amount of trait variation (57.4%), we decided to use both in subsequent analyses (see below) and hereon refer to them as fast-slow PC1 and fast-slow PC2. The PCA suggested that DTF species, all of which are deciduous, exhibit a restricted trait variation compared to the trait space of the other species. For this reason, we included interactions between the first two axes of the ‘fast-slow’ spectrum with leaf habit and biome in the initial models described below.

#### 2.5.3 Variance partitioning

To determine the relative contributions of biome, leaf habit, and species to variation in NSC for each tree organ, we fitted an intercept only linear mixed-effects model with a nested random effects structure (~1|Biome/Leaf habit/Species/Tree) using restricted maximum likelihood (REML) with the *lme* function in the R package “*nlme*” (Pinheiro et al., 2020). Variance partitioning was estimated using the *varcomp* function. The variance partitions represent the amount of variation within each hierarchical level, i.e. the variance partition for “species” represents interspecific variation (Messier et al., 2010). Note that variation within the tree level also includes residual variation, meaning that this partition captures intra-specific variation and error.

#### 2.5.4 Phylogenetic signal

We estimated the phylogenetic signal of NSC concentrations of tree organs as lambda directly from Bayesian phylogenetic hierarchical models (see details below) using the *hypothesis* function in the R package ¨*brms*¨ (Bürkner, 2017). Lambda values close to 0 indicate no phylogenetic signal while values close to 1 indicate trait evolution according to the Brownian motion evolutionary model where functional traits evolve following a random distribution (Molina-Venegas and Rodríguez, 2017).

#### 2.5.5 Phylogenetic hierarchical models

Because phylogenetically closely related species are likely to share similar trait values (Felsenstein, 1985; Freckleton et al., 2002), not accounting for phylogenetic relationships may reduce trait estimation accuracy and increase type I error rates (Li and Ives, 2017). Moreover, accounting for phylogenetic relationships in our analyses allows for direct comparisons across tree organs because different species were sampled for NSC across tree organs and the phylogenetic signal of NSC varied markedly across tree organs (see Results). We therefore fitted separate phylogenetic multi-level Bayesian models to examine variation in root, stem, branch, and leaf NSC as a function of biome, leaf habit, the ‘fast-slow’ spectrum (fast-slow PC 1, fast-slow PC 2), and two-way interactions between biome, leaf habit, and both axes of the ‘fast-slow’ spectrum. Because we did not sample evergreen species in the deciduous temperate forest, we did not include an interaction between biome and leaf habit. Initially, we included all interactions in all models; if the 95% credible intervals of interactions overlapped with zero, we re-fit models without these interactions. We opted to use hierarchical Bayesian models instead of hierarchical frequentist models because they directly estimate the degree of belief in parameter estimates, have greater flexibility in terms of selecting distribution families and, in the implementation we used with the R package ¨*brms*¨ (see details below; Bürkner, 2017), they account for correlation among observations due to relatedness by treating phylogenetic distances among species as a continuous variable, and not as a discrete one (Ellison, 2004).

As sampled trees were likely to be at their maximum height, we expected that the influence of tree height on NSC concentrations is similar across species. However, to account for the positive correlation between tree height and NSC concentrations within species (Sala and Hoch, 2009; Woodruff and Meinzer, 2011), we included tree height as a random slope in all models. This random effect’s structure captures the expected variation in the correlation between tree height and traits across species. To account for phylogenetic correlations among species, we included two random intercept terms for species: one term that models phylogenetic covariance and another term that accounts for repeated measurements and other effects that may be independent of phylogenetic relationships among species (Ives, 2018). The random effect’s structure allowed slope and intercept parameters to vary for each species. As NSC concentrations for roots, stems, branches, and leaves were not measured on all individuals, models were fit to subsets of data for each plant organ.

We fitted all models using weakly informative priors, four chains, and 1,500 burn-in samples per chain, after which 4,500 samples per chain (total post-warmup samples = 18,000) were used to calculate posterior distributions of model parameters. To reduce the number of divergent transitions, we set the ‘*adapt_delta*’ parameter within the ‘*brms*’ function to 0.99 for all models (Bürkner, 2017). All fixed effects were standardized using a z-transformation to enable comparisons across models. Model convergence was evaluated visually and by estimating ‘*Rhat*’ using the ‘*rhat*’ function, where values greater than 1 indicate that models have failed to converge. Based on a visual inspection of the distributions of NSC concentrations, we selected the distribution family by initially fitting each model twice, first with a Gaussian distribution and then with a log-normal distribution. We assessed which distribution fit better by comparing observed data to simulated data from the posterior predictive distribution using the *loo_compare* function with k-fold cross-validation (Bürkner, 2017). We therefore fitted the final models for roots and leaves with a Gaussian distribution and for stems and branches with log-normal distribution (**Supplementary Figure 3**). Additionally, we estimated a Bayesian *r*^2^ using the ‘*bayes_R2*’ function for each model to represent an estimate of the proportion of variation explained for new data.

We also compared the results of our Bayesian phylogenetic hierarchical models with those of Bayesian hierarchical models without phylogenetic correlations, which we fitted with a similar structure as described above. All analyses were performed in R version 4.3.2 (R Core Team, 2023).

## 3 Results

### 3.1 Traits and NSC concentrations across biomes and leaf habit

**Figure 1:**
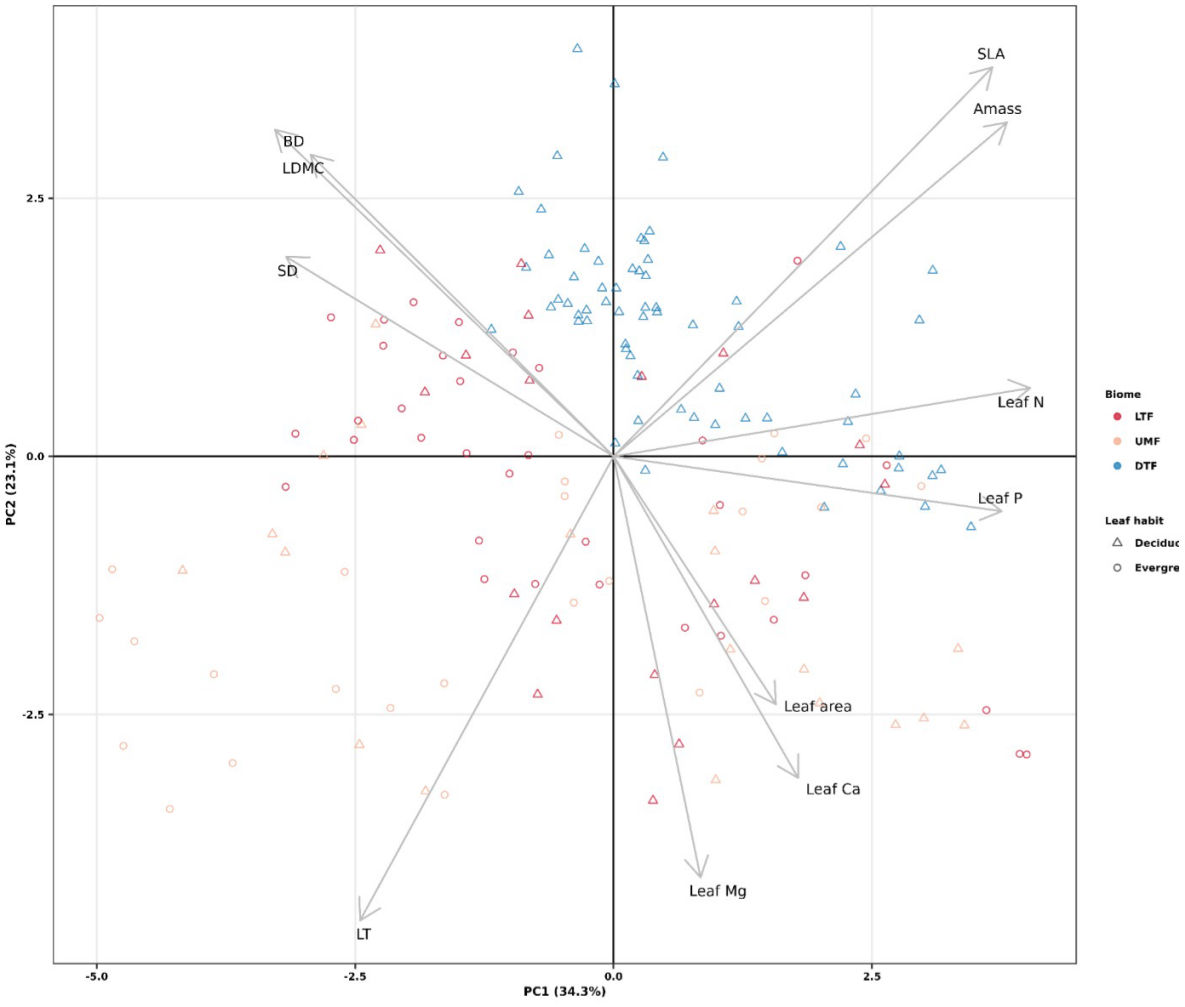
Principal components analysis of plant functional traits across biomes of tropical and temperate tree species (*n* = 61 species). LTF: lowland tropical rainforest, UMF: upper montane forest, and DTF: deciduous temperate forest. **Supplementary Table 3** shows trait abbreviations.

Functional trait values were similar across biomes except for LT, which was higher in the UMF than in LTF and DTF, and SLA, which were higher in the DTF than in the tropical biomes (95% credible intervals overlap; **Supplementary Figure 4**). Within each biome and leaf habit, tree species of the tropical biomes exhibited a broad range of variation in ecological strategies, in contrast to the DTF species, all of which are deciduous (**Figure 1**). The first two axes of the PCA captured a total of 57.4% of variation among the 11 functional traits, the first axis capturing 34.3% of variation and the second capturing 23.1%. The first fast-slow PCA (PC 1) axis represents traits associated with mechanical strength, defense, and resource acquisition, from ‘slow’ species with high branch and stem density and LDMC, to fast species with high *A*_mass_, leaf N, and leaf P. The second PCA (PC2) axis represents traits related to resource acquisition and defense, from ‘slow’ species with high leaf thickness, leaf Mg and leaf area to fast species with high SLA (**Figure 1**).

**Figure 2:**
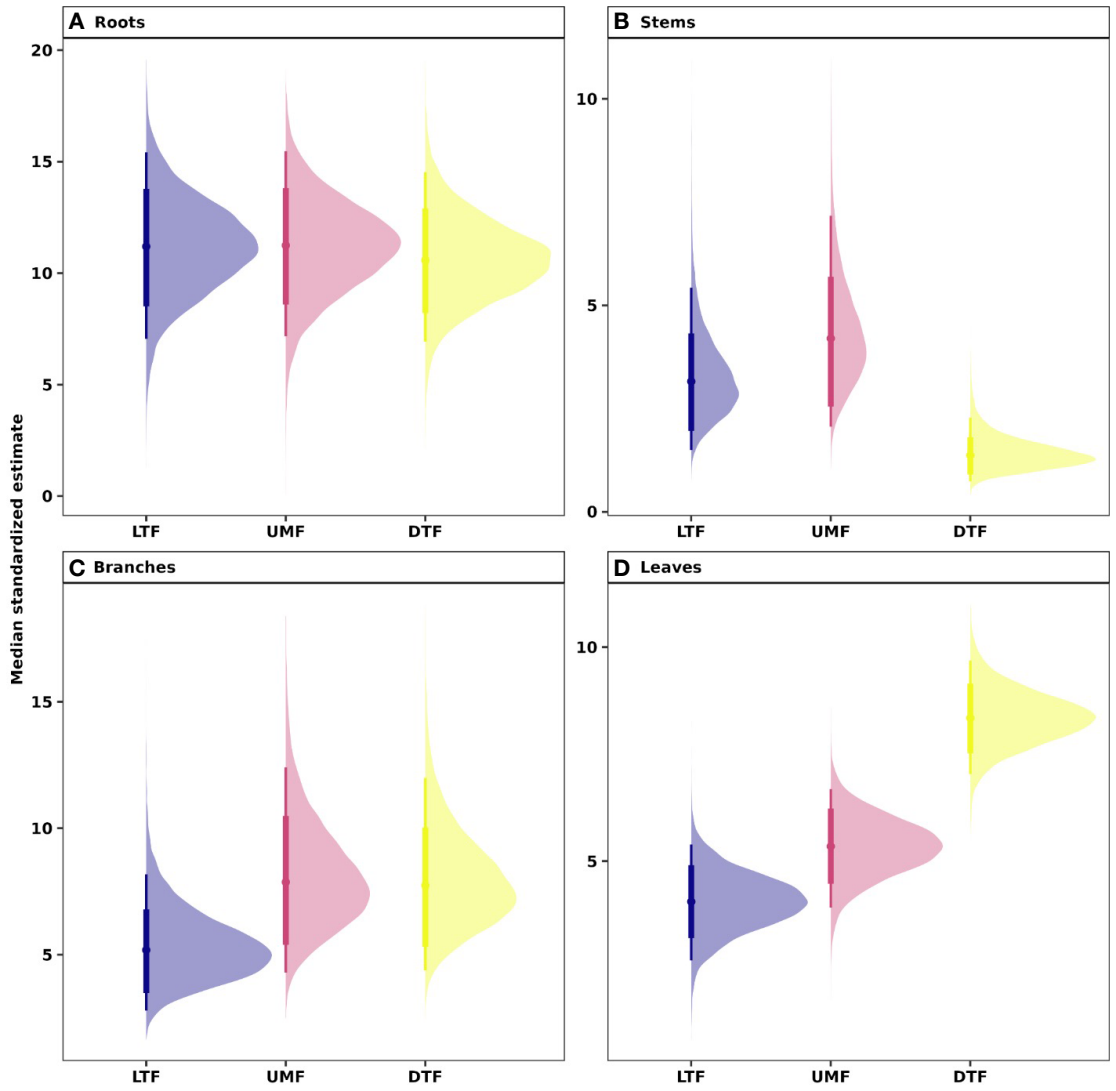
Estimated NSC concentrations in **(A)** root, **(B)** stem, **(C)** branch, and **(D)** leaves across biomes of tropical and temperate tree species. Phylogenetic hierarchical Bayesian models were fitted to estimate NSC concentrations; points are medians and whisker bars are 80% and 95% credible intervals. LTF, lowland tropical rainforest; UMF, upper montane forest; and DTF, deciduous temperate forest.

**Figure 3:**
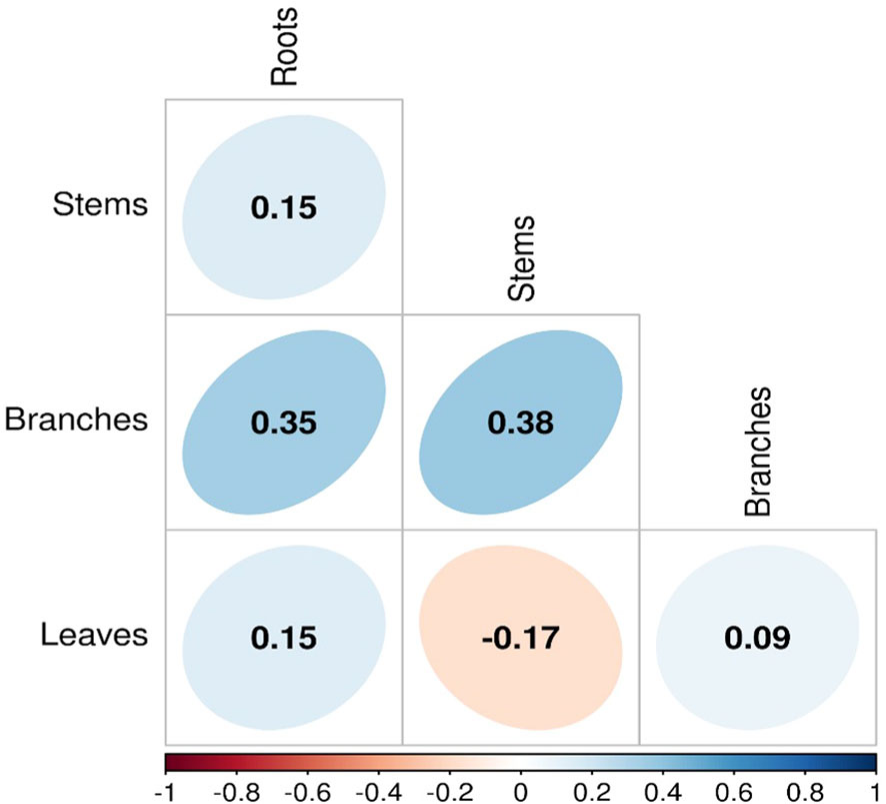
Pairwise correlations of NSC carbohydrates among tree organs across three biomes.

**Figure 4:**
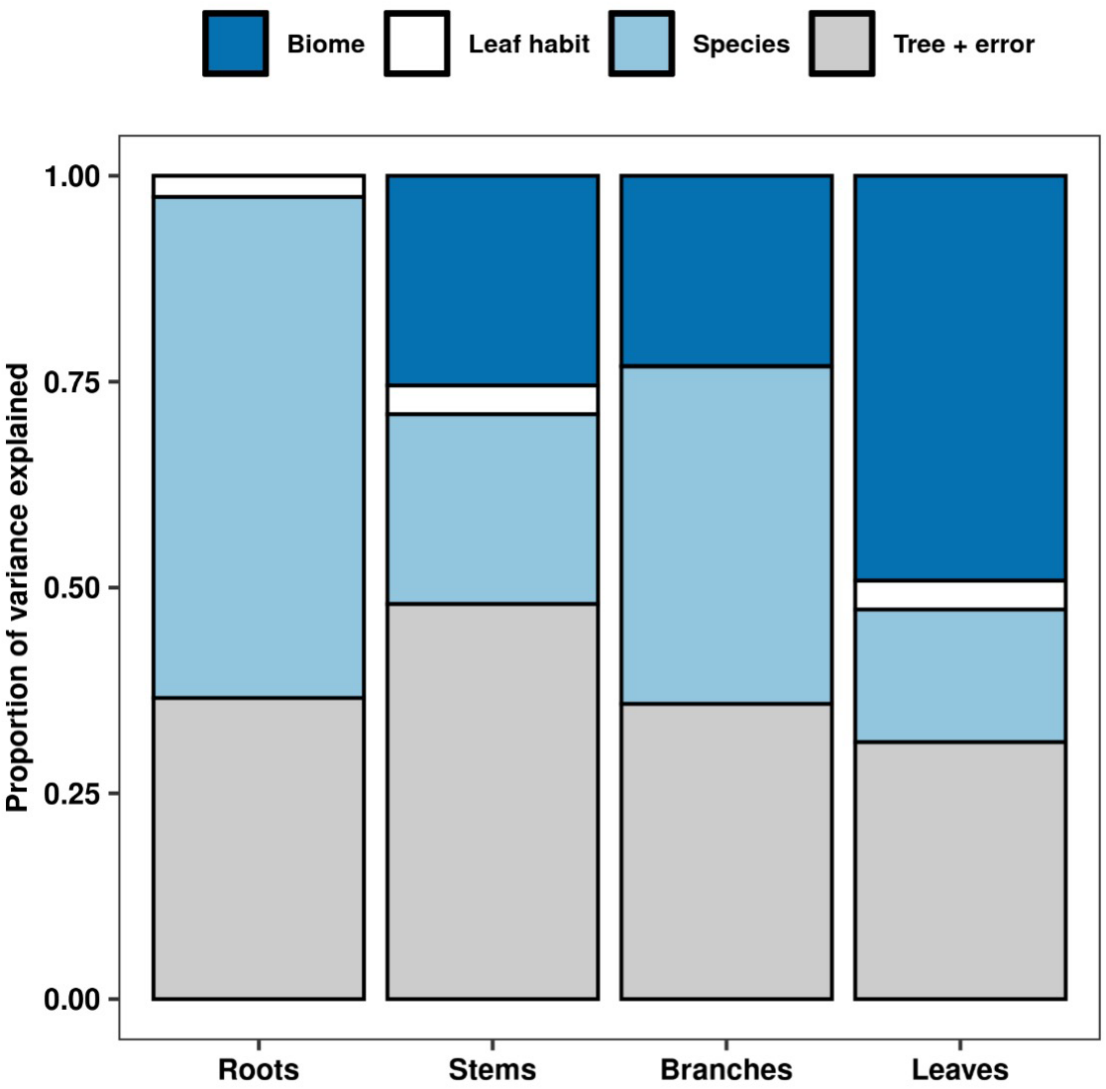
Variance partitioning of NSC concentrations for each tree organ across biome, leaf habit, and species. Variation across trees includes residual error.

Our analyses showed that NSC concentrations were similar across biomes for roots and branches, yet varied across biomes for stems and leaves (**Figure 2**). NSC concentrations of stems were lower in the DTF, while those of leaves were higher in the DTF. Among organs, however, NSC concentrations were weakly correlated (*r* < 0.4, **Figure 3**). Variance partitioning analysis showed that most of the variation in NSC in tree organs is explained by interspecific variation for roots, stems, and branches and by biomes for leaves (**Figure 4**). There was a minimal influence of leaf habit on the variation of NSC concentrations for any tree organ. The mean phylogenetic signal of NSC concentrations was highest for stems, indicating a moderate amount of phylogenetic signal but not as much as would be expected under Brownian movement (**Table 1**). Yet, the phylogenetic signal for leaves, branches and roots was close to 0, indicating that phylogenetic relatedness does not predict similarity in NSC concentrations (**Table 1**).

**Table 1:** Model fit and phylogenetic signal in NSC concentrations of organs of tropical and temperate tree species.

Organ	*r*^2^	Pagel’s lambda	Rhat
Leaves	0.66 (0.57-0.72)	0.05 (0.0 -0.29)	1.00044 (1.0004-1.00048)
Branches	0.53 (0.38-0.65)	0.08 (0.0-0.30)	1.00088 (1.00078-1.00097)
Stems	0.54 (0.34-0.69)	0.37 (0.04-0.63)	1.00045 (1.0004-1.0005)
Roots	0.72 (0.6-0.81)	0.06 (0.0-0.26)	1.00038 (1.00033-1.00044)

Model convergence (Rhat) and Pagel’s lambda were estimated directly by phylogenetic multi-level Bayesian models. 95% credible intervals are in parentheses.

### 3.2 Relationships between NSC concentrations and the fast-slow continuum across biomes and leaf habits

**Figure 5:**
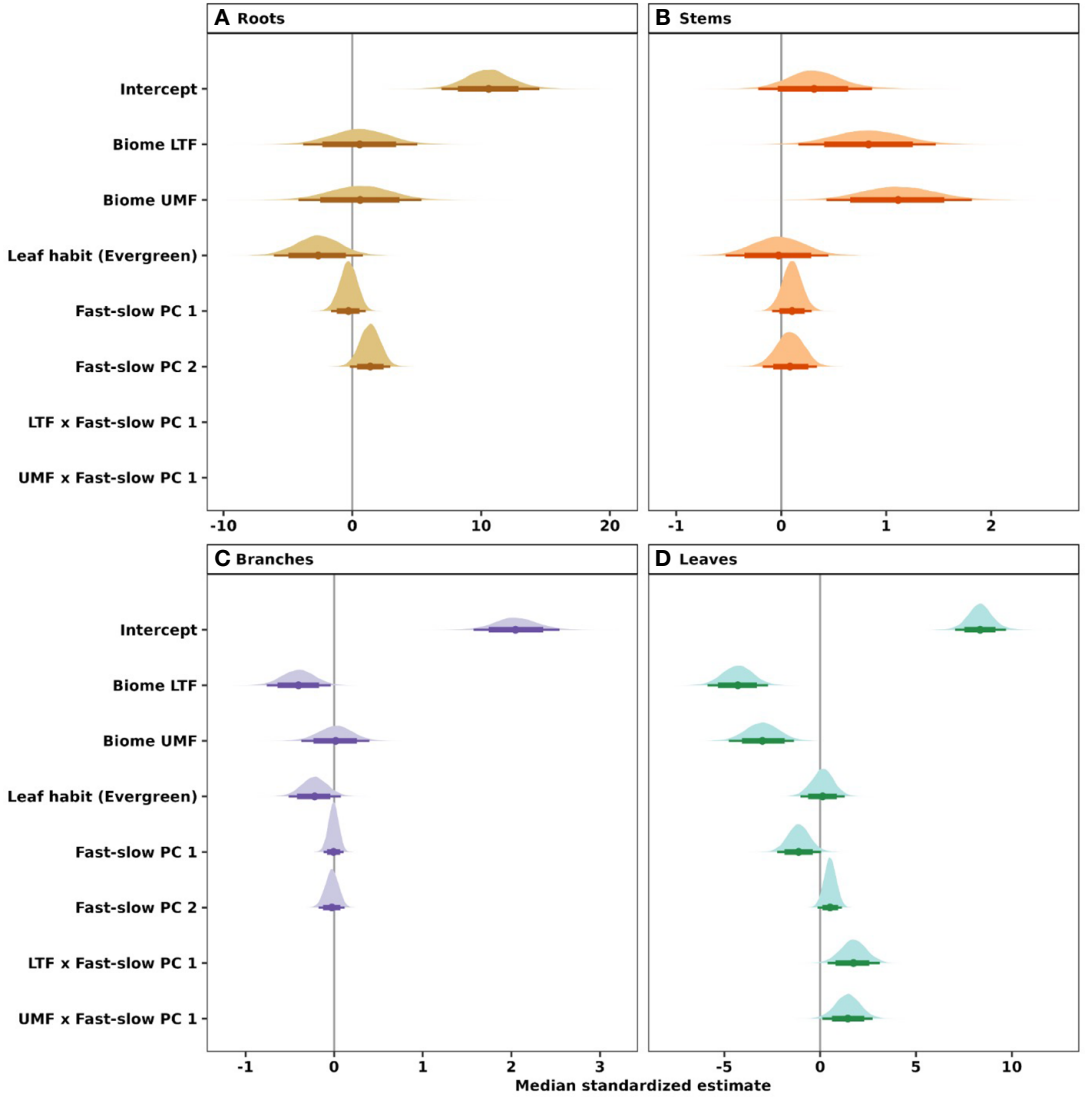
Influence of the fast-slow continuum, biomes, and leaf habit on NSC concentrations of **(A)** roots, **(B)** stems, **(C)** branches, and **(D)** leaves of tropical and temperate tree species. Phylogenetic hierarchical Bayesian models were fitted to examine variation in NSC concentration; points are medians and whisker bars are 80% and 95% credible intervals. Continuous variables were *z*-transformed prior to analysis to facilitate comparisons (within and across tree organs). Fast-slow PC 1 and fast-slow PC 2 are the first two axes of a principal component analysis of fast-slow plant functional traits. LTF, lowland tropical rainforest; UMF, upper montane forest; and DTF, deciduous temperate forest. **Supplementary Table 3** shows trait abbreviations.

Our phylogenetic multi-level Bayesian models that examine variation in root, stem, branch, and leaf NSC as a function of biome, leaf habit, and the traits of the ‘fast-slow’ spectrum (fast-slow PC 1, fast-slow PC 2), explained a large amount of variation in NSC concentrations, ranging from 53% to 72% across tree organs (mean Bayesian *r*^2^; **Table 1**) and estimated NSC concentrations (**Figure 2**). The second dimension of the ‘fast-slow’ spectrum (fast-slow PC 2) exhibits a moderate positive relationship with NSC concentrations in roots across biomes (80% credible intervals, **Figure 5A**, **Supplementary Table 4**). This indicates that more resource acquisitive species tend to have a higher concentration of reserves in roots. Fast-slow PC 1 varied negatively with leaf NSC concentrations, but the biome - fast-slow PC1 interaction varied positively with leaf NSC concentrations (**Figure 5D**, **Supplementary Table 4**). In contrast, fast-slow PC 2 exhibited a moderate positive relationship with NSC concentrations in leaves. Leaf habit had a marginally negative relationship with NSC concentrations in roots and branches (80% credible intervals, **Figures 5A, C**), indicating that evergreen species tend to have a lower NSC concentration in these tissues. The results of the Bayesian hierarchical model without phylogenetic correlations were quantitatively consistent with those of the models presented in the main text (**Supplementary Figure 5**, **Supplementary Table 5**).

**Figure 6:**
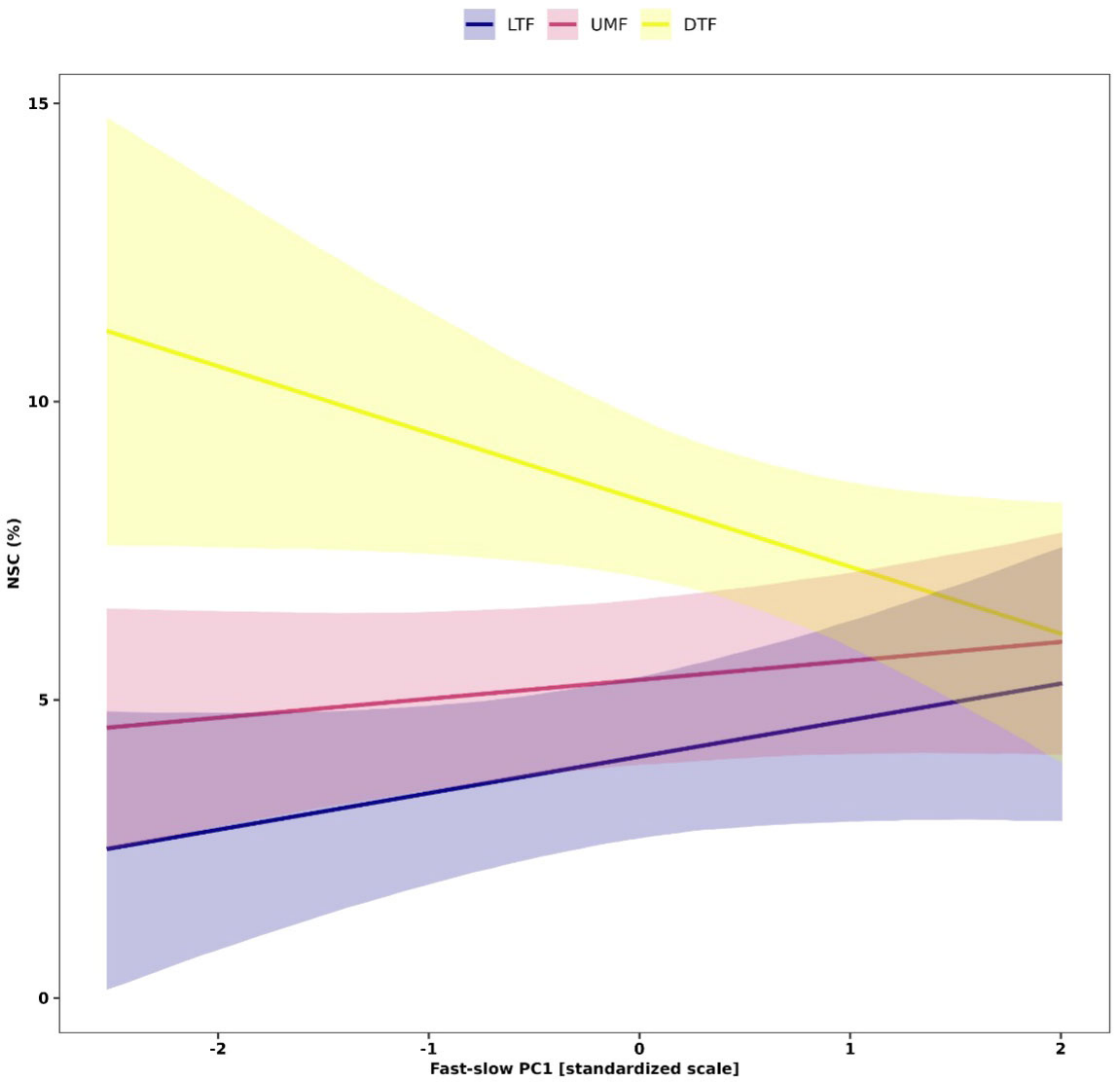
Interactive effects of the fast-slow continuum PC1 that represent the *‘*fast-slow*’* spectrum of plant form and function and biomes on NSC concentrations of leaves of tropical and temperate tree species. Solid lines are predicted fitted values from hierarchical Bayesian models and shaded regions represent 95% credible intervals of fitted values. Plant functional traits were z-transformed prior to analysis. LTF, lowland tropical rainforest; UMF, upper montane forest; and DTF, deciduous temperate forest.

Our analysis further showed context dependent effects of the ‘fast-slow’ spectrum on NSC in leaves (**Figure 6**). Leaf NSC concentrations varied slightly along the fast-slow PC1 in the tropical biomes (LTF and UMF positively), while leaf NSC varied negatively with fast-slow PC1 in the DTF (**Figure 6**). This result indicates that in the DTF, ‘slow’ species along the first fast-slow dimension have higher leaf NSC concentrations than fast species.

## 4 Discussion

Our examination of the relationships between carbon reserve concentrations and functional traits of temperate and tropical tree species revealed no coordination between traits and NSC concentrations for woody organs in stems and branches and a weak one in roots. Conversely, we found that coordination between traits and NSC concentrations for leaves was context dependent, varying markedly in direction and strength among biomes. Leaf habit did not exhibit a consistent effect on NSC concentrations across tree organs.

### 4.1 Coordination between carbohydrate concentrations and functional traits

In general, our results show that relationships between functional traits and carbohydrate concentrations in woody organs were not consistently coordinated (**Figure 5**). This suggests that the position of species along the ‘fast-slow’ plant economics spectrum is not predictive of NSC concentrations in woody organs, extending the findings from previous studies showing no trade-off between NSC concentrations and carbon investment (Lusk and Piper, 2007; Piper et al., 2009; Imaji and Seiwa, 2010; Piper, 2015) by considering species from multiple biomes.

A possible explanation for the lack of a consistent relationship between functional traits and NSC concentrations in woody organs may be that long-term allocation of carbohydrates to storage in stems or roots can take several growing seasons or years (Carbone et al., 2013; Richardson et al., 2015; Hartmann and Trumbore, 2016; Muhr et al., 2016), depending on the distance and the osmotic gradient between carbohydrate sources and sinks (Lacointe, 2000; Le Roux et al., 2001). If other trait values capture the current abiotic and biotic conditions to a greater extent than those present during the accumulation of NSC, the strength of their association with NSC concentrations may weaken with increasing age of stored NSC. Therefore, the difference in age between NSC of leaves and woody organs may explain why variation in functional traits is largely decoupled from NSC of woody organs, but not from leaf NSC.

Among the studied traits included in the fast-slow dimensions, it was surprising that variation in NSC was decoupled from wood density of stems and branches. Wood density has been suggested to be a proxy for both the amount of parenchyma (Ziemińska et al., 2015; Morris et al., 2016), and NSC concentrations (Plavcová and Jansen, 2015; Plavcová et al., 2016). However, parenchyma cells have multiple functional roles, e.g., acting as a water reservoir and contributing to different mechanical properties of wood (i.e., elasticity) that are independent of wood density and NSC concentrations (Ziemińska et al., 2015). Additionally, xylem structure – that enhances mechanical stability – places strong constraints on the storage capacity of tree stems (Plavcová et al., 2019), which may further explain why fast-slow PC1, the dimension with which SD and BD are most strongly associated, was decoupled from NSC concentrations in stems (**Figure 5**).

In contrast with NSC concentrations of woody organs, we found evidence of some coordination between leaf NSC concentrations and traits related with the ‘fast-slow’ plant economic spectrum (fast-slow PC1) (**Figure 5D**, **Supplementary Table 4**). The decrease in leaf NSC with increasing leaf N, leaf P, SLA and *A*_mass_ and the increase in leaf NSC with increasing SD, BD, and LT suggest that species with ‘slow’ ecological strategies accumulate more NSC in their leaves than those with ‘fast’ ecological strategies. Our results suggest that acquisitive trait values of leaf N, leaf P, SLA and *A*_mass_ are associated with structural support traits such as leaf toughness and leaf lifespan (Osnas et al., 2018). However, the evidence for the coordination between trait- leaf NSC concentrations was found to be moderate, as only 80% credible intervals did not overlap with zero. Finally, even though leaf N and P are strongly correlated with *A*_mass_ (Reich and Schoettle, 1988) because physiologically they play a fundamental role in both photosynthesis and starch and sucrose synthesis (Rychter et al., 2016), they do not seem to favor NSC accumulation in woody structures in temperate and tropical biomes, at least in stem and branches.

Thus, our results did not fully support our second hypothesis that species with ‘slow’ traits associated with greater carbon investment in defense and conservative ecological strategies accumulate more NSC in woody organs than species associated with acquisitive or ‘fast’ ecological strategies. Our results show that species with higher NSC concentrations in roots exhibit a weak tendency to have trait values associated with ‘fast’ but not ‘slow’ ecological strategies. This finding suggests that ‘fast’ species may allocate more carbohydrates to roots than ‘slow’ species as part of their response to mechanical damage to aboveground plant organs, which may enable them to persist in areas subjected to frequent disturbances, such as winds, low intensity fires (Poorter et al., 2010; Clarke et al., 2013), ice storms (Proulx and Greene, 2001), or in human-dominated ecosystems (Uhl, 1987; Jakovac et al., 2015). Among the few cases where traits predicted variation in NSC, we found contrasting trends in trait-NSC relationships among biomes, especially in leaves. Leaf NSC did not vary or increased with fast-slow PC 1 in the tropical biomes, but decreased towards ‘fast’ species along the ‘fast-slow’ trait spectrum in the DTF (**Figure 6**). Several studies on woody plants have reported contrasting patterns of plant functional strategies among biomes, which have been associated with phylogenetic constraints, or selective biogeographic processes, such as adaptation to different climatic regimes or physical barriers that generate different selective pressures within communities (Wright et al., 2005; Heberling and Fridley, 2012; 2013; Zanne et al., 2014). Additionally, other abiotic factors, such as soil fertility and water availability, may mediate the growth-storage trade-off for NSCs by either facilitating or constraining tree growth (Breugel et al., 2011).

### 4.2 Patterns of NSC storage across tree organs

While plants can remobilize nutrients and reserves between organs according to fluctuating resource availability (Maillard et al., 2015), our results show that trees accumulate large amounts of carbohydrate reserves over time in woody organs – especially in roots – regardless of their ecological strategy or leaf habit (**Figure 2**). The high NSC concentrations in roots observed in this study may indicate that roots serve as the long-term reservoir for responding to future disturbances (Clark and Clark, 1991; Poorter et al., 2010; Clarke et al., 2013), ensuring that resources are available for resprouting or leaf flush (Wiley, 2013). For example, multiple studies from multiple forest biomes have shown that trees have sufficient reserves to rebuild the entire leaf canopy up to four times (Hoch et al., 2003; Körner, 2003; Würth et al., 2005; Herrera-Ramírez et al., 2021) or provide the carbon necessary for stem growth for up to 30 years (Klein et al., 2016; D’Andrea et al., 2019). Thus, NSC stored in stems and roots probably remain stable or increase gradually over time, at least until a severe disturbance triggers an imbalance between carbon sources and sinks and initiates mobilization of reserves. The stability of root and stem NSC reserves likely differs from the more labile, more recently produced NSC reserves stored in leaves and branches that support daily metabolism and annual growth (Martínez-Vilalta et al., 2016). Examining storage dynamics of starch and lipids, and the extent to which they are coordinated with the ‘fast-slow’ plant economics spectrum or response traits may deepen our understanding of ecological strategies that underpin interspecific variation in tree growth and mortality (Herrera-Ramírez et al., 2021).

## 5 Conclusions

Our study tested the hypothesis that NSC concentrations in tree organs are associated with the ‘fast-slow’ spectrum of leaf and wood functional traits across biomes. In woody organs, we only found a moderate positive relationship with NSC concentrations in roots across biomes. Considering the concentrations of NSC in woody organs as a proxy for species’ capacity to respond to disturbances, our results imply that variation in species’ NSC concentrations is weakly associated with functional trait spectra that describe global variation in plant life history. Consequently, efforts to predict the response of ecosystems to global change will need to integrate a suite of response traits that are independent of the ‘fast-slow’ spectrum and that capture species’ resilience to global change drivers.

## Data availability statement

The datasets presented in this study can be found in online repositories. The names of the repository/repositories and accession number(s) can be found below: All data and code used in this article is available in Github: https://github.com/dylancraven/Traits_NSC/tree/main.

## Author contributions

JAR: Conceptualization, Formal analysis, Methodology, Visualization, Writing – original draft, Writing – review & editing, Data curation, Investigation, Resources. DC: Data curation, Formal analysis, Investigation, Methodology, Visualization, Writing – original draft, Writing – review & editing. DH: Formal analysis, Writing – review & editing. JP: Conceptualization, Writing – original draft. BR: Conceptualization, Data curation, Formal analysis, Writing – original draft. CS: Conceptualization, Methodology, Data curation, Writing – original draft. GH: Conceptualization, Data curation, Methodology, Writing – original draft. IH: Conceptualization, Data curation, Formal analysis, Investigation, Methodology, Writing – original draft, Funding acquisition, Resources, Writing – review & editing. CM: Conceptualization, Data curation, Formal analysis, Funding acquisition, Investigation, Methodology, Writing – original draft, Writing – review & editing, Project administration, Resources.
