# Supplementary material for: Non-structural carbohydrate concentrations in tree organs vary across biomes and leaf habits, but are independent of the fast-slow plant economic spectrum
Source: Front Plant Sci. 2024 May 3;15:1375958. doi: 10.3389/fpls.2024.1375958 (PMC11099217; doi:10.3389/fpls.2024.1375958)
Supplement: Supplementary file 1 [file DataSheet_1.docx]

Supplementary Material

Non-structural carbohydrate concentrations in tree organs show variations among biomes and leaf habits and no to small dependence to the fast-slow plant economic spectrum

**Ramirez, J.A.^*^, Craven, D., Herrera, D.A., Posada, J.M., Reu, B., Sierra, C.A., Hoch, G., Handa, I.T., and Messier, C.**

*** Correspondence:** Jorge Andres Ramirez: [j.ramirez@unicauca.edu.co](mailto:j.ramirez@unicauca.edu.co)

# Supplementary Figures and Tables

##
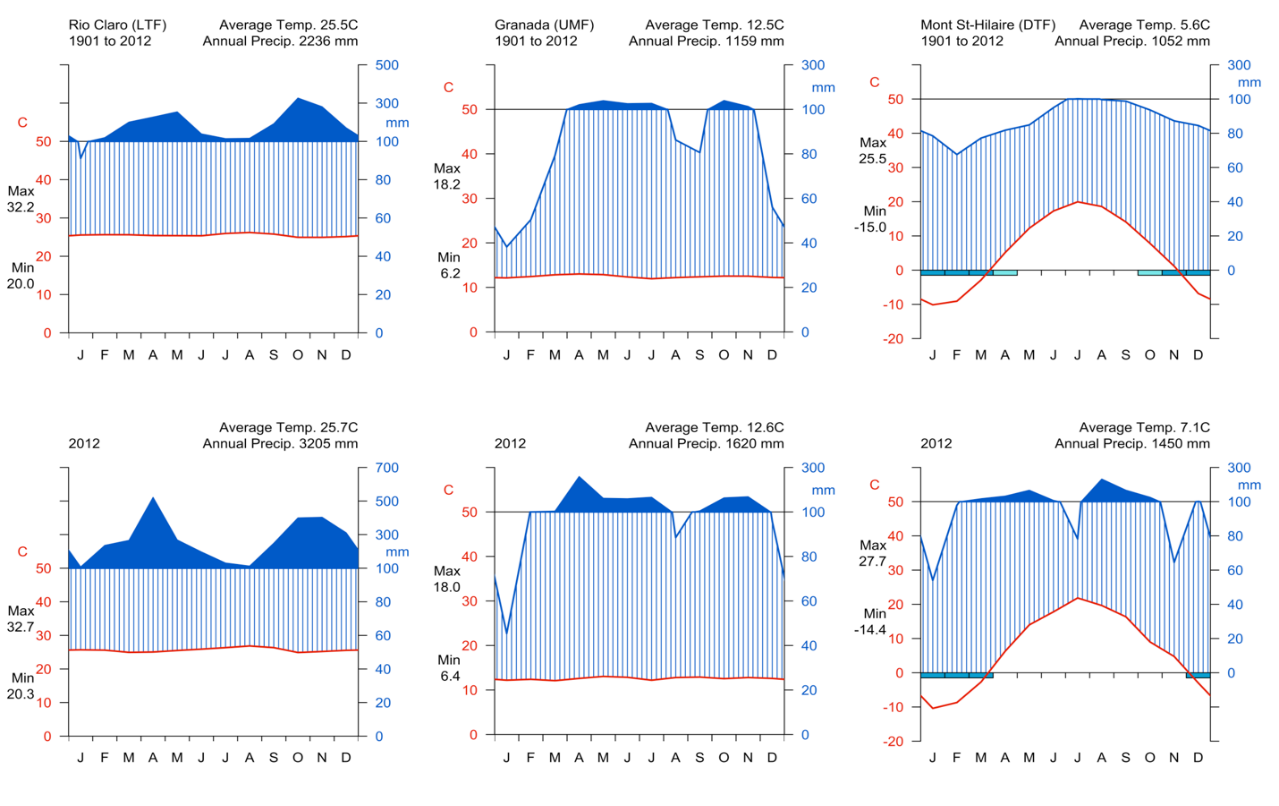
Supplementary Figures

**Supplementary Figure 1.** Walter-Lieth climate diagrams of the three study sites. Data from high-resolution gridded dataset of the Climatic Research Unit (CRU) at the University of East Anglia (Harris et al. 2014). In the first row, average climate data from 1901 to 2012 and in the second row, average climate data since 2012. UMF: upper montane tropical forest, LTF: lowland tropical rainforest, and DTF: deciduous temperate forest.


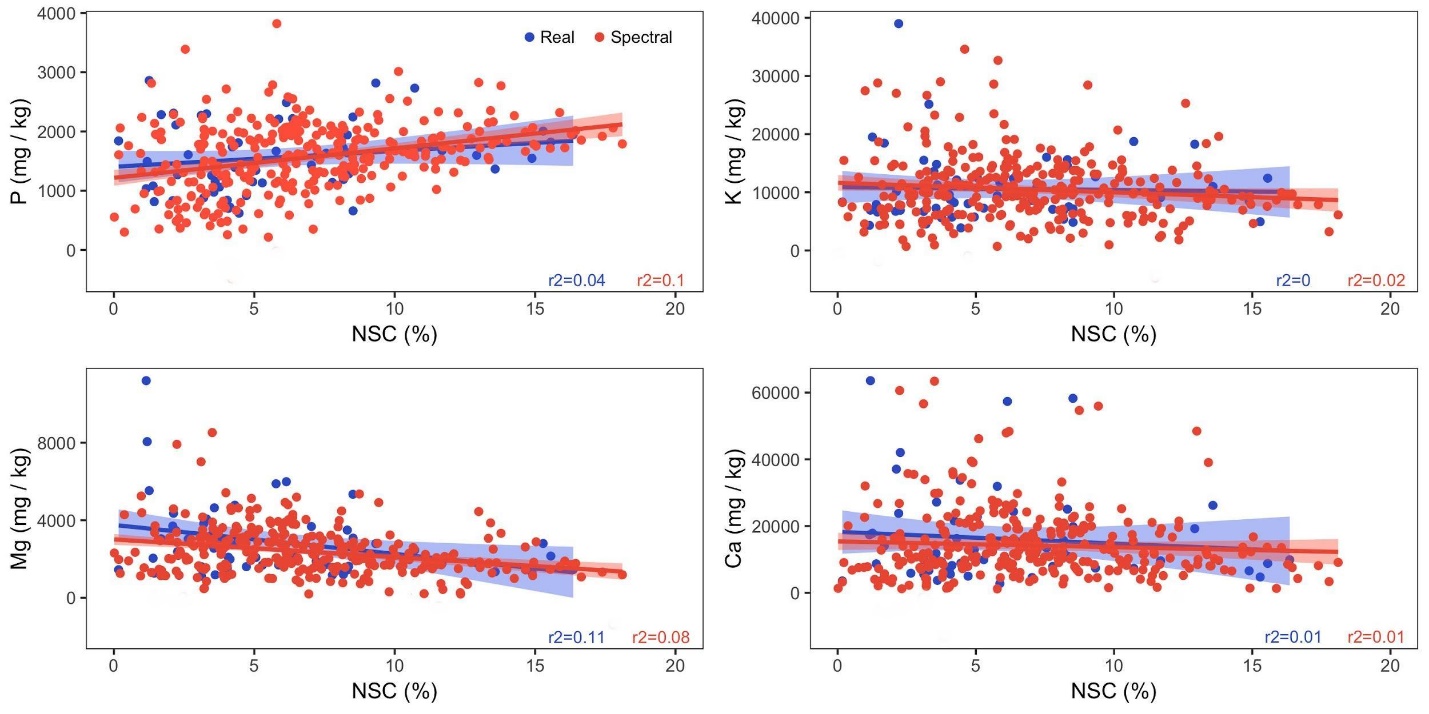
**Supplementary Figure 2.** Relationships between direct measures and imputed spectral values of leaf NSC and functional traits. Refer to Table S3 for trait abbreviations.


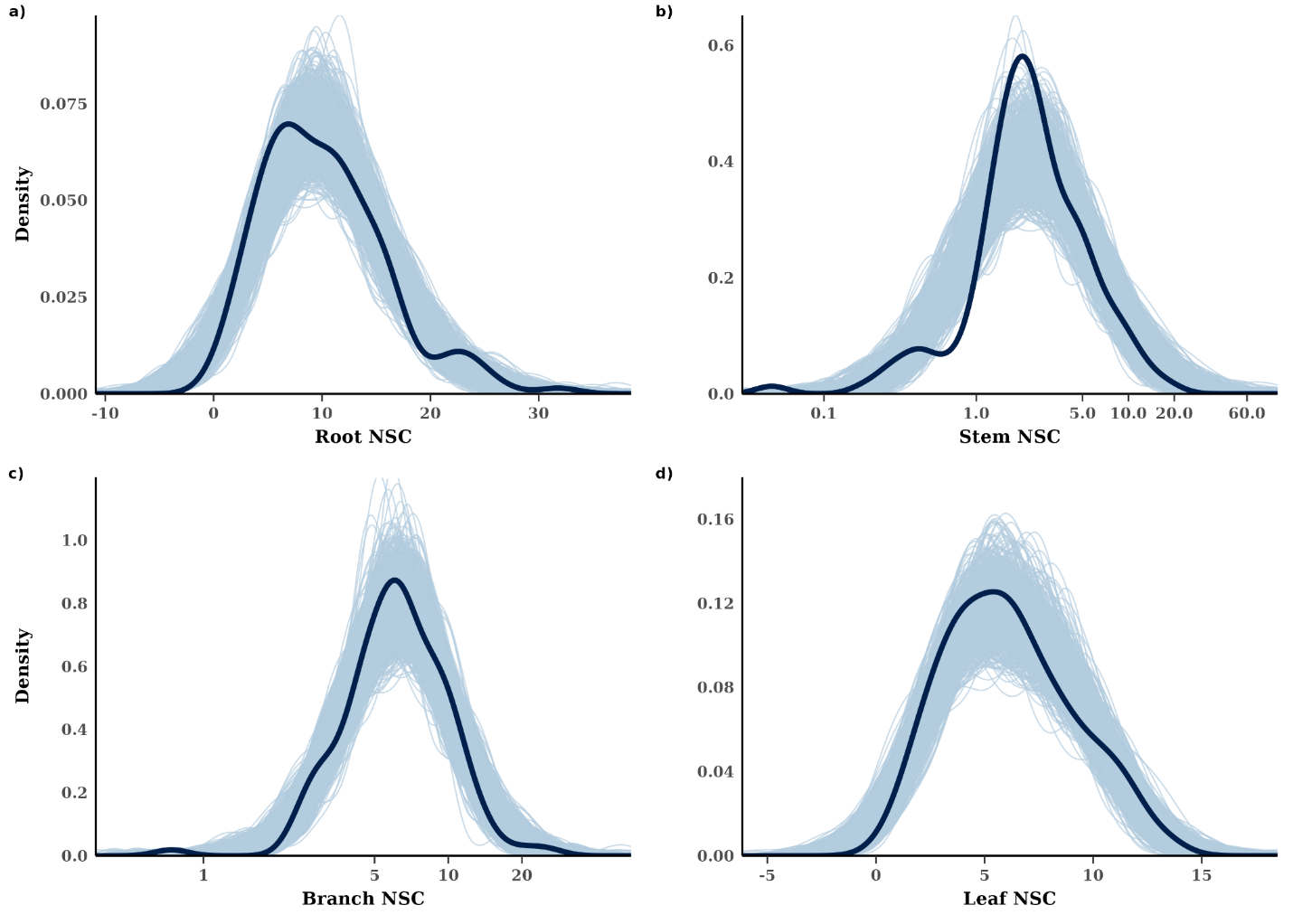
**Supplementary Figure 3**. Posterior predictive distributions for non-structural carbohydrates of (A) roots, (B) stems, (C) branches, and (D) leaves estimated by phylogenetic hierarchical Bayesian models. Dark blue lines represent the observed data, while the light blue lines represent simulations of the response variable ( n = 1,000 posterior samples).

**
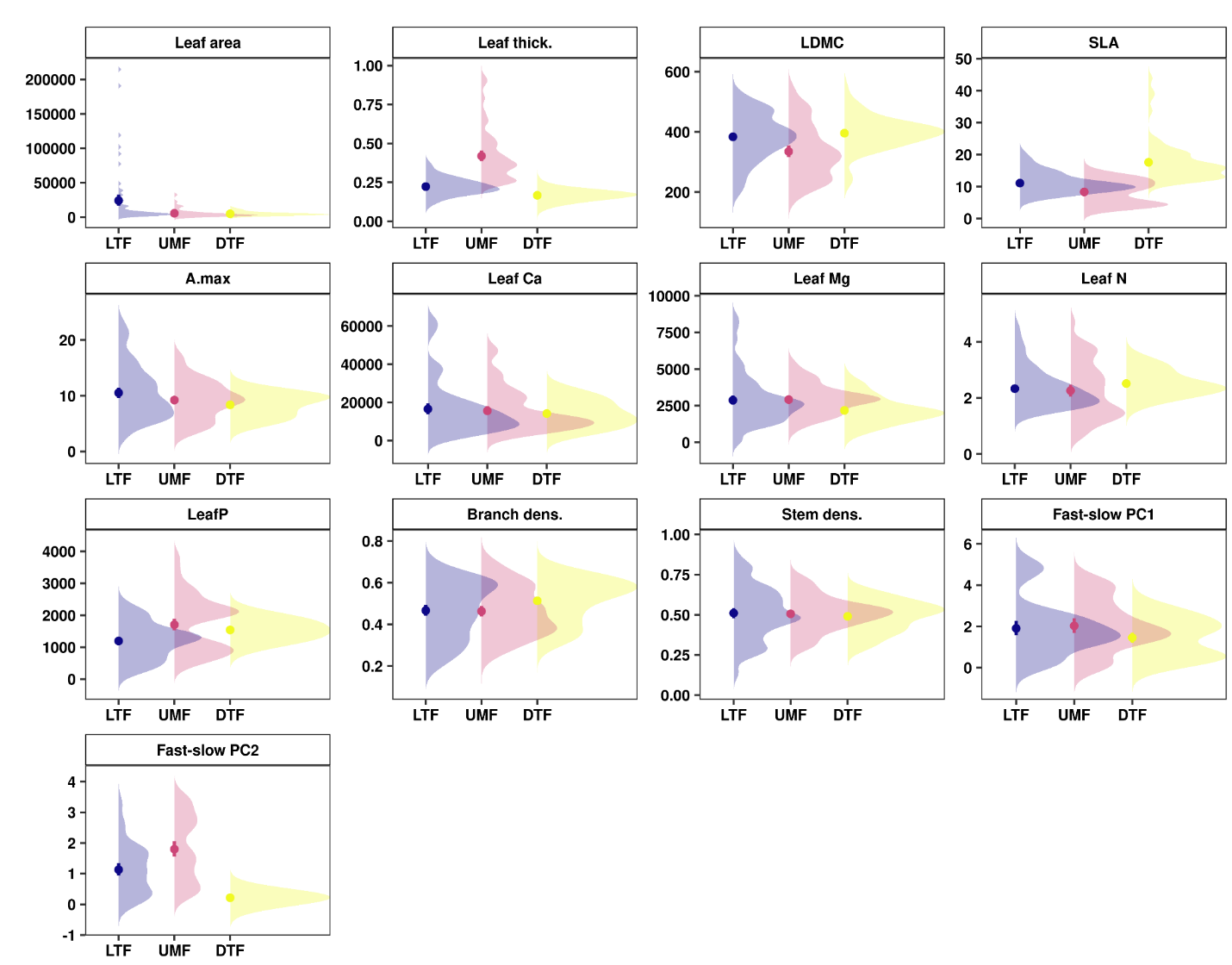
Supplementary Figure 4.** Trait means and distributions of plant functional traits and fast-slow PCs across biomes. Whisker bars show means and 95% confidence intervals. LTF: lowland tropical rainforest, UMF: upper montane forest, and DTF: deciduous temperate forest. Table S3 shows trait abbreviations.


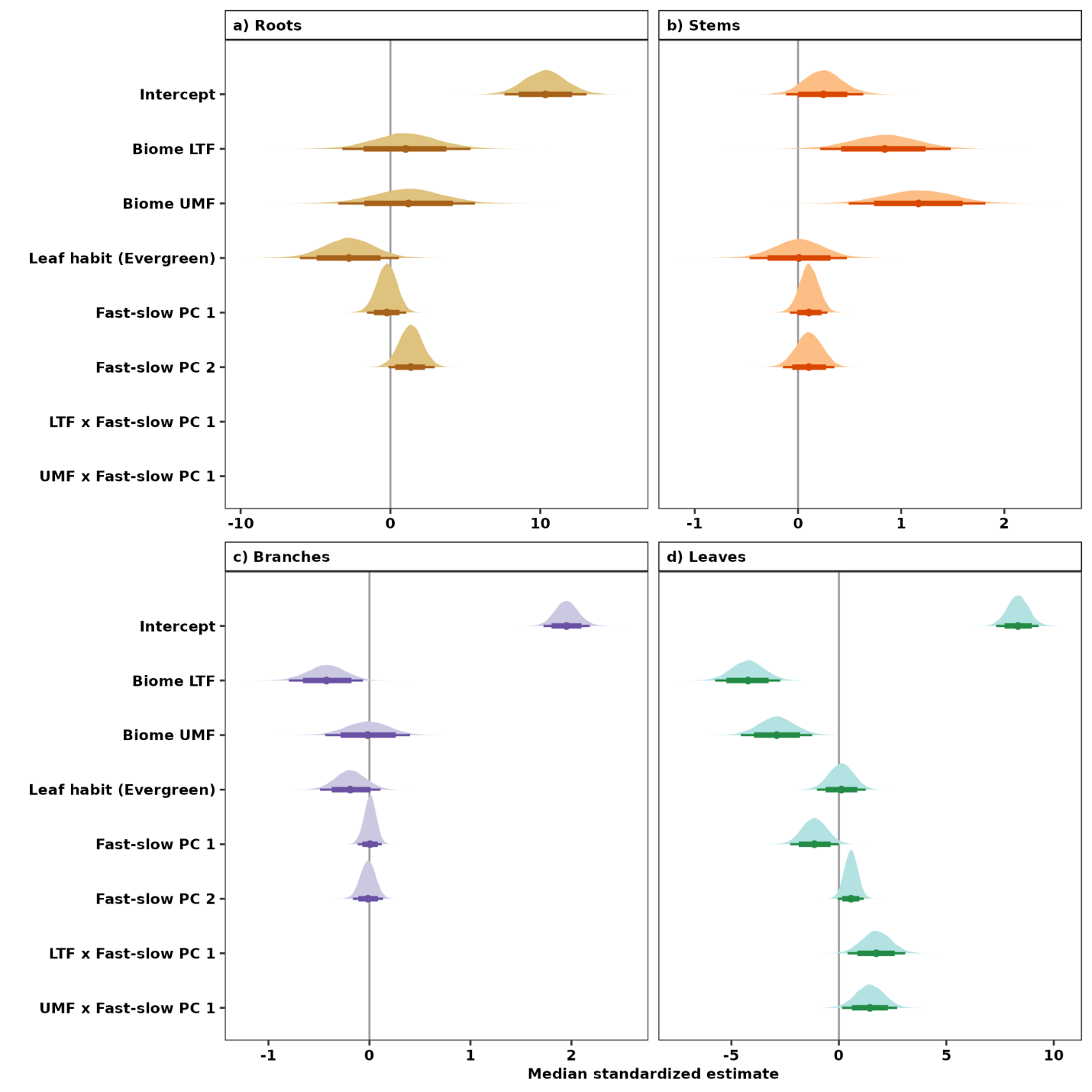


**Supplementary Figure 5.** Influence of the fast-slow continuum, biomes, and leaf habit on NSC concentrations of (A) roots, (B) stems, (C) branches, and (D) leaves estimated by non-phylogenetic hierarchical Bayesian models. Points are medians and whisker bars are 80% and 95% credible intervals. Continuous variables were *z*-transformed prior to analysis to facilitate comparisons (within and across tree organs). Fast-slow PC1 and fast-slow PC2 are the first two axes of a principal component analysis of fast-slow plant functional traits. of LTF: lowland tropical rainforest, UMF: upper montane forest, and DTF: deciduous temperate forest. Table S3 shows trait abbreviations.

## Supplementary Tables

**Supplementary Table 1.** Main characteristics of the study sites.

|  | Rio Claro Reserve Antioquia, Colombia (LTF biome) | Hacienda Sabaneta Nature Reserve  Cundinamarca, Colombia (UMF biome) | Gault Nature Reserve Mont-Saint-Hilaire Quebec, Canada (DTF biome) |
| --- | --- | --- | --- |
| **Biome** | Lowland tropical rainforest | Upper montane forest | Deciduous temperate forest |
| **Latitude** | 5°54’04’’ N | 4°32’30’’ N | 45°32’31’’ N |
| **Longitude** | 74°51’24’’ W | 74°15’18’’ W | 73°09’11’’ W |
| **Altitude (range, m asl)** | 250 – 750 | 2500 – 3300 | 200 – 400 |
| **Mean annual precipitation (mm)** | 3882† | 1956† | 967‡ |
| **Precipitation regime** | Bimodal | Bimodal | Unimodal |
| **Mean annual temperature (°C)** | 26 | 12 | 6 (16*) |
| **Mean annual freeze-free days** | 0 | 0 | 140‡ |
| **Soil order** | Entisol | Andisol | Chernozemic |
| **Number of species studied** | 32 | 27 | 21 |
| **Disturbance history** | Reserve protected since 1976 | Reserve protected since 2008 | Reserve protected since 1958 |

* Mean growing season temperature.

† IDEAM (Institute of Hydrology, Meteorology and Environmental Studies of Colombia).

‡ Environment and natural resources. Canada.

**Supplementary Table 2**. List of tree species sampled in Colombia and Canada along with its leaf habit. UMF: upper montane tropical forest, LTF: lowland tropical rainforest, and DTF: deciduous temperate forest.

| **Species** | **Family** | **Biome** | **Leaf habit** |
| --- | --- | --- | --- |
| *Acer pensylvanicum* | Sapindaceae | DTF | Deciduous |
| *Acer rubrum* | Sapindaceae | DTF | Deciduous |
| *Acer saccharum* | Sapindaceae | DTF | Deciduous |
| *Alnus acuminata* | Betulaceae | UMF | Evergreen |
| *Alnus rugosa* | Betulaceae | DTF | Deciduous |
| *Apeiba glabra* | Malvaceae | LTF | Deciduous |
| *Aspidosperma megalocarpon* | Apocynaceae | LTF | Evergreen |
| *Bellucia pentamera* | Melastomataceae | LTF | Evergreen |
| *Betula alleghaniensis* | Betulaceae | DTF | Deciduous |
| *Betula papyrifera* | Betulaceae | DTF | Deciduous |
| *Brosimum utile* | Moraceae | LTF | Evergreen |
| *Cariniana pyriformis* | Lecythidaceae | LTF | Deciduous |
| *Casearia arborea* | Salicaceae | LTF | Evergreen |
| *Cavendishia bracteata* | Ericaceae | UMF | Evergreen |
| *Cecropia peltata* | Urticaceae | LTF | Evergreen |
| *Cedrela montana* | Meliaceae | UMF | Deciduous |
| *Cespedesia spathulata* | Ochnaceae | LTF | Deciduous |
| *Citharexylum poeppigii* | Verbenaceae | UMF | Evergreen |
| *Clathrotropis brachypetala* | Fabaceae | LTF | Evergreen |
| *Clusia multiflora* | Clusiaceae | UMF | Evergreen |
| *Cordia alliodora* | Boraginaceae | LTF | Deciduous |
| *Varronia cylindrostachya* | Boraginaceae | UMF | Deciduous |
| *Cordia spp.* | Boraginaceae | UMF | Deciduous |
| *Croton killipianus* | Euphorbiaceae | LTF | Deciduous |
| *Drimys granadensis* | Winteraceae | UMF | Evergreen |
| *Duguetia antioquensis* | Annonaceae | LTF | Evergreen |
| *Fagus grandifolia* | Fagaceae | DTF | Deciduous |
| *Fraxinus americana* | Oleaceae | DTF | Deciduous |
| *Goupia glabra* | Goupiaceae | LTF | Deciduous |
| *Hieronyma alchorneoides* | Phyllanthaceae | LTF | Evergreen |
| *Hymenaea courbaril* | Leguminosae | LTF | Evergreen |
| *Ilex nervosa* | Aquifoliaceae | UMF | Evergreen |
| *Iryanthera megistocarpa* | Myristicaceae | LTF | Evergreen |
| *Juglans cinerea* | Juglandaceae | DTF | Deciduous |
| *Juglans neotropica* | Juglandaceae | UMF | Deciduous |
| *Lecythis ampla* | Lecythidaceae | LTF | Deciduous |
| *Miconia biappendiculata* | Melastomataceae | UMF | Evergreen |
| *Morella parvifolia* | Myricaceae | UMF | Deciduous |
| *Muntingia calabura* | Muntingiaceae | LTF | Evergreen |
| *Myrsine coriacea* | Primulaceae | UMF | Evergreen |
| *Ochoterenaea colombiana* | Anacardiaceae | LTF | Evergreen |
| *Ochroma pyramidale* | Malvaceae | LTF | Evergreen |
| *Oreopanax bogotensis* | Araliaceae | UMF | Deciduous |
| *Ostrya virginiana* | Betulaceae | DTF | Deciduous |
| *Piper bogotense* | Piperaceae | UMF | Evergreen |
| *Populus grandidentata* | Salicaceae | DTF | Deciduous |
| *Populus tremuloides* | Salicaceae | DTF | Deciduous |
| *Protium aracouchini* | Burseraceae | LTF | Evergreen |
| *Prunus buxifolia* | Rosaceae | UMF | Deciduous |
| *Prunus serotina* | Rosaceae | DTF | Deciduous |
| *Pseudoxandra sclerocarpa* | Annonaceae | LTF | Evergreen |
| *Quercus rubra* | Fagaceae | DTF | Deciduous |
| *Solanum humboldtianum* | Solanaceae | UMF | Evergreen |
| *Handroanthus guayacan* | Bignoniaceae | LTF | Deciduous |
| *Tapirira guianensis* | Anacardiaceae | LTF | Evergreen |
| *Tilia americana* | Malvaceae | DTF | Deciduous |
| *Trema micrantha* | Cannabaceae | LTF | Evergreen |
| *Ulmus americana* | Ulmaceae | DTF | Deciduous |
| *Verbesina crassiramea* | Compositae | UMF | Deciduous |
| *Viburnum lasiophyllum* | Viburnaceae | UMF | Evergreen |
| *Vismia macrophylla* | Hypericaceae | LTF | Evergreen |

**Supplementary Table 3.** List of functional traits considered in this study with the abbreviations used in the text, the units of expression, and the hypothesized ecological function associated with the trait (Baraloto *et al.* 2010; Fortunel, Fine & Baraloto 2012).

| **Parameter** | **Abbreviation** | **Units** | **Ecological role** |
| --- | --- | --- | --- |
| Leaf size | LS | mm^2^ | Resource acquisition |
| Leaf thickness | LT | Mm | Resource acquisition and defense |
| Leaf dry matter content | LDMC | mg g^-1^ | Resource acquisition and defense |
| Specific leaf area | SLA | mm^2^ mg^-1^ | Resource acquisition and defense |
| Photosynthetic capacity | Amass | nmol CO_2_ g^-1^ s^-1^ | Resource acquisition |
| Foliar nitrogen | Leaf N | % | Resource acquisition and defense |
| Foliar phosphorus | Leaf P | mg kg^-1^ | Resource acquisition |
| Foliar calcium | Leaf Ca | mg kg^-1^ | Resource defense |
| Foliar magnesium | Leaf Mg | mg kg^-1^ | Resource acquisition and defense |
| Stem density | SD | mg mm^-3^ | Hydraulic transport, mechanical strength and defense |
| Branch density | BD | mg mm^-3^ | Hydraulic transport, mechanical strength and defense |
| Tree height | H | M | Resource capture and reproduction |
| Sugars | Sugar | % | Carbon and energy source |
| Starch | Starch | % | Carbon and energy source |
| NSC | NSC | % | Carbon and energy source |

**Supplementary Table 4.** Summary of phylogenetic hierarchical Bayesian models that examine the variation in NSC concentrations of roots, stems, branches, and leaves.

|  | **Roots** | | **Stems** | | **Branches** | | **Leaves** | |
| --- | --- | --- | --- | --- | --- | --- | --- | --- |
| Fixed effect | Estimate | Effective sample size | Estimate | Effective sample size | Estimate | Effective sample size | Estimate | Effective sample size |
| Intercept | 10.61 (6.93 - 14.53) | 8421 | 0.32 (-0.18 - 0.91) | 8681 | 2.05 (1.57 - 2.54) | 8837 | 8.35 (7.05 - 9.72) | 12200 |
| Biome LTF | 0.57 (-3.81 - 5.03) | 6082 | 0.83 (0.18 - 1.49) | 7988 | -0.41 (-0.78 - -0.05) | 9572 | -4.30 (-5.89 - -2.74) | 11158 |
| Biome UMF | 0.58 (-4.28 - 5.28) | 6704 | 1.12 (0.43 - 1.81) | 7742 | 0.01 (-0.38 - 0.39) | 9187 | -3.02 (-4.71 - -1.30) | 11682 |
| Leaf habit (Evergreen) | -2.66 (-6.14 - 0.78) | 7923 | -0.03 (-0.51 - 0.47) | 10413 | -0.22 (-0.52 - 0.07) | 10880 | 0.12 (-1.05 - 1.27) | 13967 |
| Fast-slow PC1 | -0.30 (-1.69 - 1.01) | 7586 | 0.1 (-0.08 - 0.29) | 13363 | -0.01 (-0.12 - 0.10) | 11538 | -1.12 (-2.25 - 0.05) | 11329 |
| Fast-slow PC2 | 1.38 (-0.19 - 2.93) | 10684 | 0.08 (-0.18 - 0.34) | 7122 | -0.03 (-0.18 - 0.12) | 10733 | 0.52 (-0.12 - 1.14) | 14947 |
| LTF x Fast-slow PC1 |  |  |  |  |  |  | 1.73 (0.36 - 3.08) | 13062 |
| UMF x Fast-slow PC1 |  |  |  |  |  |  | 1.44 (0.12 - 2.75) | 11613 |

The effective sample size reports the number of independent samples with the same estimation power as the N autocorrelated samples (Kruschke 2015).

**Supplementary Table 5**. Model fit and convergence of non-phylogenetic hierarchical Bayesian models that examine variation in NSC concentrations of tropical and temperate tree species. 95% credible intervals are in parentheses.

| **Organ** | ***r*^2^** | **Rhat** |
| --- | --- | --- |
| Roots | 0.72 (0.6-0.8) | 1.00068 (1.00057-1.00079) |
| Stems | 0.52 (0.32-0.69) | 1.00062 (1.00053-1.00071) |
| Branches | 0.51 (0.35-0.65) | 1.00049 (1.00041-1.00056) |
| Leaves | 0.65 (0.57-0.72) | 1.00041 (1.00035-1.00046) |
